# Bionic vision technologies: progress and perspectives on retinal prostheses and optogenetics for the treatment of advanced retinal degeneration

**DOI:** 10.3389/fmedt.2026.1898982

**Published:** 2026-08-25

**Authors:** Na Long, Meng Si, Yin Jiang, Fan Chen, Bei Chen, Fei Jiang, Shangang Pan, Xueqin Chu, Huarong Wu

**Affiliations:** 1 Department of Ophthalmology, Anqing Municipal Hospital, Anqing, China; 2 Department of Pharmacy, Anqing Municipal Hospital, Anqing, China; 3 Department of Ophthalmology, Anqing Shihanbin Aier Eye Hospital, Anqing, China

**Keywords:** advanced retinal degeneration, encoding–biointerface, neural adaptation, optogenetics, retinal prosthesis, vision restoration

## Abstract

Advanced degenerative retinal diseases [e.g., retinitis pigmentosa (RP) and age-related macular degeneration (AMD)] are characterized by irreversible photoreceptor loss, and conventional interventions rarely prevent progression to profound vision loss at advanced stages. Retinal prostheses and optogenetic therapies are two leading bionic strategies to bypass absent photoreceptors, yet both are limited by a shared “encoding–biointerface” bottleneck: converting external scenes into interpretable neural activity in surviving retinal circuits with adequate selectivity, safety margins, and long-term stability. This review critically compares the two approaches in spatial resolution, neural adaptation, and biosafety, while explicitly distinguishing clinical evidence from preclinical and proof-of-concept studies. Retinal prostheses provide near-term light perception and limited form vision but are constrained by current spread, non-selective pathway/axon recruitment, interface reactions, and cortical decoding burden. Optogenetic restoration offers cell-type–biased reactivation and potentially more physiological processing, but currently remains coupled to external light-delivery hardware and is limited by scattering, heterogeneous transduction, safety-limited irradiance, restricted dynamic range, and adeno-associated virus (AAV)–related inflammatory risk. We highlight emerging integrative directions, including opto-electronic hybrid interfaces, adaptive encoding, broadband subretinal nanomaterial-based photovoltaic nanoprostheses, and experimental acoustic modalities (e.g., focused ultrasound and sonogenetics), alongside ethical and accessibility considerations for translation.

## 1 Introduction

Advanced retinal degenerative diseases, such as retinitis pigmentosa (RP) and age-related macular degeneration (AMD), are leading causes of irreversible blindness worldwide. Their core pathological feature is the progressive degeneration and loss of photoreceptors in the outer nuclear layer (ONL). Although many inner retinal neurons [e.g., retinal ganglion cells (RGCs) and bipolar cells] can survive into late stages, degeneration is frequently accompanied by pathological retinal remodeling, including aberrant synaptic reorganization and reduced synaptic transmission efficiency, thereby compromising intrinsic retinal information processing (1–3). The extent of inner-retinal preservation and remodeling varies across disease etiologies and stages, and this heterogeneity can critically influence the achievable outcomes of vision-restoration interventions. Therefore, cross-study comparisons and “one-size-fits-all” expectations should be approached with caution, particularly when patient selection and disease stage differ. Importantly, this disease-stage dependence means that “bypassing photoreceptors” is not a uniform engineering problem, but a moving biological target in which the same interface may perform differently across patients and over time.

Because conventional pharmacological and surgical interventions rarely reverse end-stage photoreceptor loss, “bionic vision” strategies have been developed to bypass photoreceptors and stimulate surviving retinal circuits. Two principal approaches have entered clinical trials or early clinical use: retinal prostheses, which deliver electrical stimulation via implanted microelectrode arrays, and optogenetics, which uses viral vector–mediated gene transfer to confer photosensitivity on inner retinal cells (4, 5). Clinical studies show that pioneering retinal prostheses (e.g., Argus II and Alpha-AMS) enabled basic light perception and limited form vision (6, 7). However, their real-world clinical trajectory was also constrained by factors including high device and service costs, limited daily-life utility for some recipients, and the withdrawal of commercial support for certain first-generation platforms (8–10). This gap between technical feasibility and sustainable deployment highlights that translational success depends as much on long-term service ecosystems as on device performance.

Furthermore, from a technical perspective, effective resolution is profoundly constrained by electrode density and the limited selectivity of extracellular stimulation, including current spread that recruits off-target neural elements (11, 12). In parallel, optogenetics can provide cell-type–biased reactivation through opsin expression (e.g., Chronos) and patterned optical stimulation. Yet, its clinical translation remains limited by irradiance-dependent safety margins, potential interference with residual native responses, and practical constraints such as optical scattering and heterogeneous transgene expression (13–17). Moreover, remodeling-related reductions in bipolar-cell synaptic output may further limit the transmission of optogenetically evoked signals to higher visual centers (3). Taken together, these limitations suggest that the central challenge is not simply “adding pixels” or “adding photosensitivity”, but ensuring that external scenes are encoded into physiologically interpretable retinal signals through a stable and safe biointerface.

In this review, we compare retinal prostheses and optogenetics in terms of principles, clinical outcomes, and key bottlenecks, with an emphasis on how degeneration-induced circuit remodeling shapes the efficacy of interventions (18, 19). We also discuss emerging, early-stage directions—including hybrid optoelectronic interfaces, adaptive encoding concepts, sonogenetics-based ultrasound neuromodulation, and nanoparticle-mediated near-infrared approaches—as forward-looking strategies rather than clinically established solutions (12, 15, 20–22). Throughout, we explicitly distinguish clinical evidence from preclinical findings, engineering proof-of-concept studies, and perspective statements to support rigorous interpretation of the field, and we use the “encoding–biointerface” concept as an integrative lens to connect stimulation physics, tissue response, and the demands of cortical decoding.

## 2 Retinal prostheses: from electronic chips to visual perception

### 2.1 Technical principles and the evolution of anatomical implantation sites

**Figure 1:**
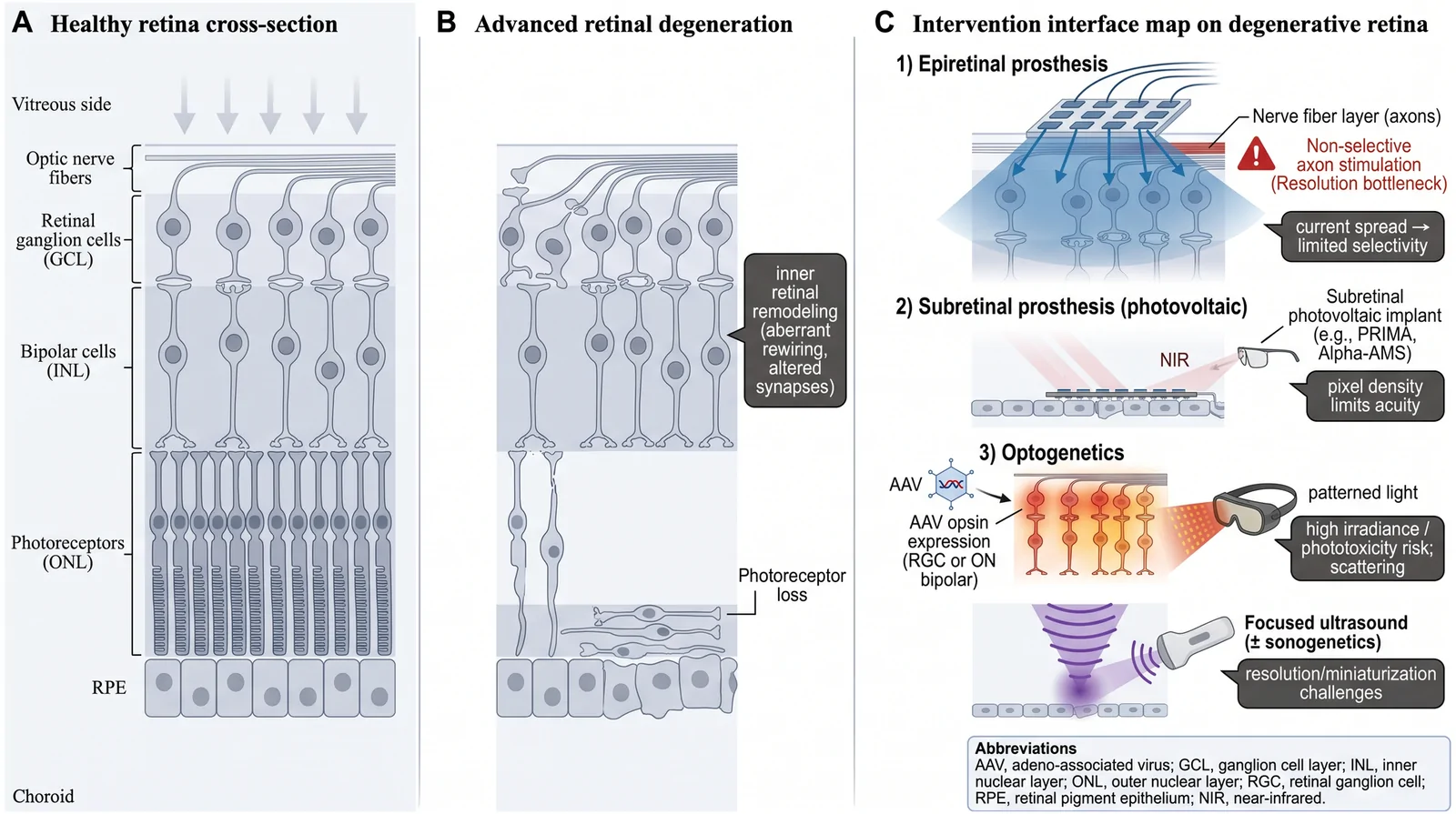
Degenerative retina and intervention targets. Schematic comparison of retinal lamination in health [Panel **(A)**] and advanced degeneration [Panel **(B)**, photoreceptor loss with inner-retinal remodeling], and an interface map of major bionic vision strategies on the degenerative retina [Panel **(C)**]. Epiretinal prostheses stimulate the inner retinal surface but are prone to non-selective recruitment of nerve fiber layer axons, a key bottleneck in resolution. Subretinal photodiode/photovoltaic implants are placed in the outer retinal space. They can be driven by near-infrared (NIR) projection in some systems. In contrast, optogenetics uses adeno-associated virus (AAV)-mediated opsin expression in surviving inner retinal cells (e.g., retinal ganglion cells or ON bipolar cells) combined with patterned light stimulation. Focused ultrasound (±sonogenetics) is shown as a non-invasive modality with current limitations in achievable resolution and miniaturization. (Schematic generated with the assistance of Gemini 3.1 image editor.). AAV, adeno-associated virus; GCL, ganglion cell layer; INL, inner nuclear layer; ONL, outer nuclear layer; RGC, retinal ganglion cell; RPE, retinal pigment epithelium; NIR, near-infrared.

The core mechanism of a retinal prosthesis is to establish an artificial visual–neural interface: visual information captured by an external camera is processed by an encoding unit and delivered to an implanted stimulator (e.g., a microelectrode array or a photovoltaic subretinal chip), which generates patterned electrical stimulation to activate surviving retinal neurons (primarily RGCs and, depending on the device, bipolar cells), thereby bypassing damaged photoreceptors and eliciting percepts in higher visual centers (8). This approach primarily targets patients with RP and related conditions in which photoreceptor loss is irreversible, whereas portions of the inner retina remain anatomically present, albeit variably remodeled. In terms of device evolution, implantation strategies have expanded to include both epiretinal (e.g., Argus II) and subretinal (e.g., Alpha-IMS/Alpha-AMS and PRIMA) placements, reflecting distinct engineering trade-offs in surgical access, stimulation selectivity, and interface stability. Because late-stage retinal degeneration can preserve inner retinal neurons while disrupting normal circuitry, the efficacy of intervention depends strongly on the anatomical interface layer and the resulting spread and selectivity of stimulation. The retinal layers affected by advanced degeneration and the interface targets of major bionic vision strategies are summarized in Figure 1.

Taking Argus II—the first “bionic eye” approved by the US Food and Drug Administration (FDA)—as an example, early implantation studies demonstrated basic safety and restoration of light perception; however, long-term follow-up revealed risks of mechanical migration and rotation of the epiretinal array (9, 23). Such positional changes can alter the electrode–retinal gap, thereby affecting stimulation thresholds and functional outcomes (24). To mitigate some physical contact–related issues and to reposition stimulation closer to the outer-retinal space, subretinal systems (e.g., PRIMA) place the photovoltaic chip in the layer previously occupied by photoreceptors. This anatomical placement may better align with residual inner-retinal processing in selected etiologies, but the effective distance to target cells can still be increased by degenerative changes in the subretinal space (e.g., debris and scarring), and epiretinal and subretinal devices also differ in their predominant neural elements recruited during stimulation (11, 25–27). Accordingly, “implant location” should be interpreted as a trade-off among target recruitment, selectivity, and disease-dependent anatomy rather than a simple hierarchy of “more physiological” vs. “less physiological”. In addition, PRIMA employs subthreshold photovoltaic technology and uses near-infrared (NIR) light for wireless power delivery and signal transmission, which can reduce the need for transcutaneous leads and may also minimize interference with residual visible-light perception in patients with atrophic AMD (11, 25).

In addition to epiretinal and subretinal placements, the suprachoroidal route constitutes a third anatomical implantation strategy, advanced by the Bionic Vision Australia consortium (with key contributions from Petoe and colleagues) (28–30). By positioning the electrode array in the suprachoroidal space—between the sclera and choroid—this approach potentially reduces surgical invasiveness and retinal traction compared with epiretinal implants, while avoiding the need for retinotomy or subretinal bleb formation required in subretinal approaches (30, 31). First-in-human studies demonstrated the feasibility of this approach with an acceptable safety profile, although mild subretinal hemorrhage was observed as an expected postoperative event that resolved spontaneously without sequelae (30). Subsequently, a second-generation 44-channel suprachoroidal prosthesis was evaluated in a clinical trial, showing significant improvements in functional vision, activities of daily living, and observer-rated quality of life over 2.7 years of follow-up, with no device-related serious adverse events (28, 29). However, stimulation thresholds are generally higher and spatial resolution remains limited relative to subretinal counterparts, reflecting the increased electrode–retina distance inherent to this anatomical placement (28, 29). These trade-offs underscore that suprachoroidal implantation should be interpreted as a strategic compromise between surgical safety and stimulation efficiency, rather than a universally superior alternative (31).

### 2.2 Clinical outcomes and technical limitations

Although retinal prostheses represent an important clinical advance—enabling basic light perception and coarse object localization in individuals with profound blindness—major limitations remain in restoring high-resolution, naturalistic vision (8). First, spatial resolution is limited and heterogeneous. Clinical follow-up indicates substantial inter-individual variability in visual benefit: across various retinal prostheses, only a subset of participants demonstrated marked improvement in basic localization tasks, whereas others showed little benefit, and in some complex tasks (e.g., motion discrimination), performance often failed to exceed chance levels (24, 28, 29). Such variability suggests that reported benefits are highly endpoint- and context-dependent, and cohort-level averages may mask substantial responder–nonresponder differences. From a mechanistic perspective, limited spatial fidelity reflects both physical constraints (electrode density and current spread) and physiological constraints of extracellular stimulation, including non-selective recruitment of multiple retinal pathways and, in epiretinal devices, a tendency to activate RGC axons in the nerve fiber layer, which can distort perceived phosphene location and shape (11, 12). This implies that rehabilitation can only partially compensate for a stimulus that is physiologically ambiguous at the retinal output level.

Second, long-term biocompatibility at the tissue–electrode interface can deteriorate. Chronic implantation may induce glial proliferation and fibrotic encapsulation, leading to progressive increases in electrode impedance and reduced charge-injection efficiency; in addition, local electrode corrosion and stimulation-related thermal effects may contribute to chronic inflammatory responses (32). Meta-analyses suggest that implant-related serious adverse events (e.g., array displacement and conjunctival erosion) can occur throughout long-term follow-up and constitute key factors limiting device longevity (33).

Third, challenges related to neural adaptation and cross-modal compensation persist, particularly for earlier-generation epiretinal systems. Because the spatiotemporal characteristics of prosthesis-evoked percepts can differ fundamentally from those of natural vision, the brain may not readily decode these inputs. For some platforms, recipients require extensive practice to interpret intermittent percepts as meaningful contours, and many users have difficulty with rapidly changing dynamic stimuli (34). In complex real-world environments, prosthesis-derived vision alone is often insufficient, and many recipients appear to rely on auditory–visual cross-modal mapping to support spatial search (34). Notably, training requirements and the immediacy of task performance can differ across device types and patient populations; for example, PRIMA users have been reported to read letters shortly after device activation under appropriate testing conditions, although performance may still improve with continued use and task practice (35). Therefore, training burden should be reported platform-by-platform rather than generalized from early epiretinal systems to newer subretinal designs.

### 2.3 High-density arrays, flexible materials, and closed-loop intelligence

**Figure 2:**
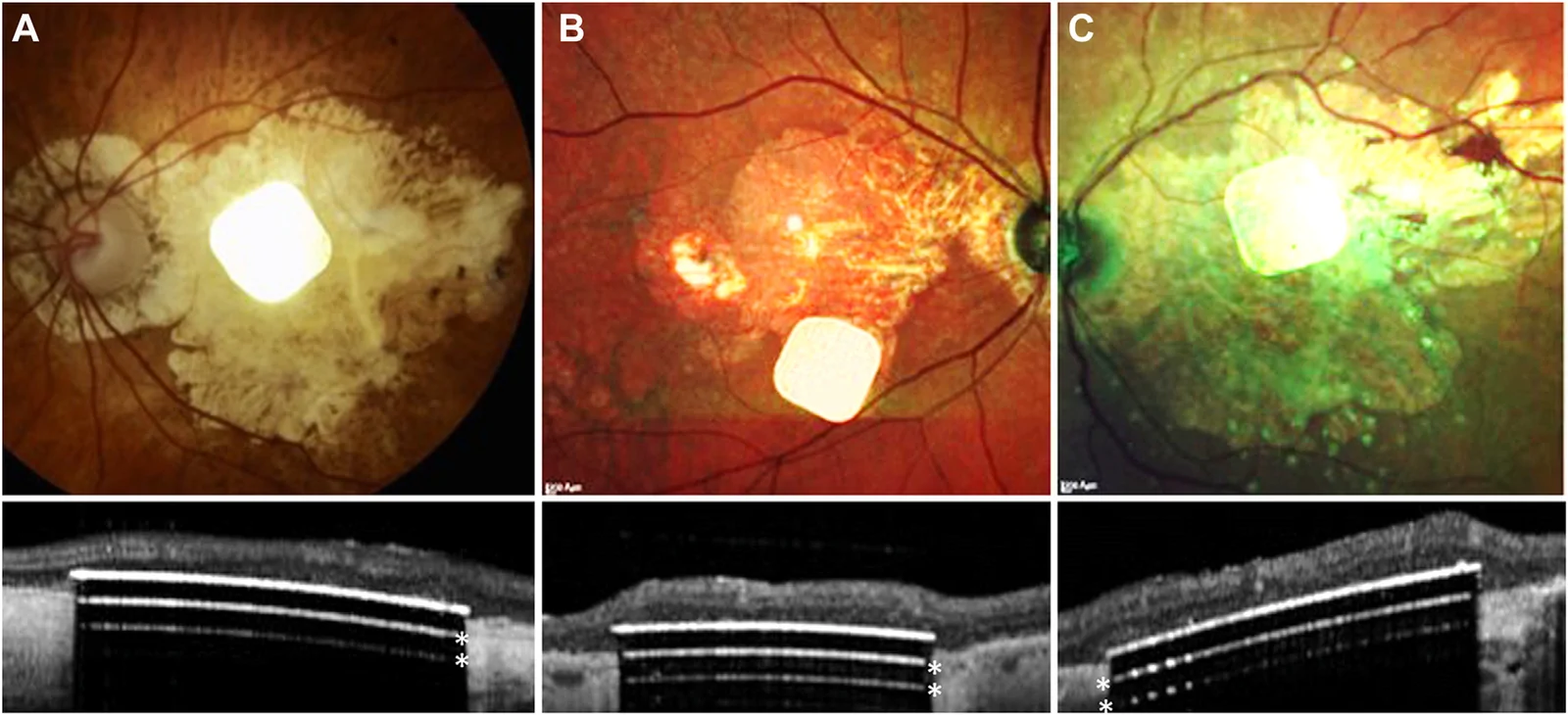
Real-world clinical imaging of PRIMA subretinal implant positioning at long-term follow-up (fundus photography and OCT) (25). **(A–C)** Real-world clinical fundus photographs (top row) and corresponding OCT B-scans (bottom row) at 48 months after implantation of a PRIMA subretinal microchip in three patients with geographic atrophy due to age-related macular degeneration. The fundus photographs show the implant footprint within the atrophic region, while OCT confirms subretinal positioning of the implant relative to the RPE/Bruch's complex. Two bright linear signals beneath the implant surface (marked with asterisks) represent OCT artifacts due to strong reflections from the implant surface and should be distinguished from true anatomical layers when interpreting postoperative imaging. [Reprinted from (25), under CC BY-NC-ND 4.0.]. AMD, age-related macular degeneration; GA, geographic atrophy; OCT, optical coherence tomography; PRIMA, photovoltaic retinal implant microarray; RPE, retinal pigment epithelium.

To address the above clinical bottlenecks, recent studies have focused on innovations in hardware materials and optimization of software algorithms. For higher-density stimulation, the PRIMA system uses a micrometer-scale photovoltaic array (378 pixels; 100 µm per pixel). In clinical reports, native (no-zoom) prosthetic visual acuity measured without electronic magnification has been reported as approximately 20/500 in earlier follow-up. More recent updates report a mean native (no-zoom) visual acuity of 20/420. At the same time, performance can be further enhanced by electronic zoom at the cost of a reduced effective field of view (25, 35). Critically, zoom-assisted task gains should not be conflated with native prosthetic acuity when comparing resolution across platforms. Representative long-term fundus photography and optical coherence tomography (OCT) B-scans illustrate the stable subretinal position of PRIMA implants and their relationship to the underlying retinal pigment epithelium (RPE). Notably, strong reflections from the implant surface can introduce OCT artifacts, which should be considered when interpreting postoperative imaging. Representative long-term *in vivo* fundus and OCT imaging of PRIMA implants is shown in Figure 2.

Meanwhile, a new-generation 256-channel epiretinal prosthesis [Intelligent Micro Implant Eye (IMIE) 256] demonstrated improved grating acuity and motion discrimination compared with earlier devices (e.g., the 60-channel Argus II) in a first-in-human study (36). However, higher channel or pixel counts do not guarantee proportional functional gains if current spread and changes at the biological interface limit selectivity. Regarding flexible materials, to reduce mechanical injury and lower impedance, flexible electrodes based on liquid metals (e.g., eutectic gallium–indium alloys) have been shown to conform closely to retinal curvature and increase the charge-injection limit (37). In addition, carbon nanotube modification can reduce microelectrode interface impedance by approximately 50% (from 17 *Ω* to 8 *Ω* at 1 kHz), suggesting improved durability for long-term neural interfacing (38). To mitigate electrical crosstalk in high-density flexible polymer arrays, investigators have used equivalent-circuit modeling and finite element analysis to optimize electrode spacing and insulation thickness (39).

Beyond material advances, increased intelligence and closed-loop control represent another important trend. Bidirectional closed-loop systems can monitor electrode–tissue impedance and evoked responses from RGCs or the cortex in real time, enabling adaptive adjustment of stimulation parameters to reduce the risk of overstimulation and support tissue safety (11, 40). At the software level, retinal prosthesis–oriented biomimetic edge-detection algorithms can selectively reduce the number of pixels that require activation while maintaining task-relevant visual cues, thereby lowering operating power consumption in high-density arrays (41). In parallel, emerging subretinal nanomaterial-based photovoltaic nanoprostheses—such as tellurium nanowire network implants enabling bias-free broadband (visible-to-infrared) photocurrent generation—have restored light-evoked responses and improved vision-guided behaviors in blind mice and elicited retina-derived responses in non-human primates, highlighting an experimental route toward higher sensitivity without bulky auxiliary power delivery (preclinical) (42). Collectively, these advances are pushing retinal prostheses toward higher-density stimulation and more adaptive biointerfaces, while persistent constraints from stimulation selectivity, tissue response, and long-term stability remain central translational challenges. The technical specifications, anatomical placements, and reported clinical outcomes of representative systems are summarized in Table 1.

**Table 1:** Specifications and clinical outcomes of representative retinal prosthetic systems.

System	Placement	Key hardware features	Clinical/preclinical outcomes & notes	Ref
Argus II	Epiretinal	60-Ch MEA	Acuity-related endpoint ∼20/1,260; high variance; distorted percepts.	(10–12)
Alpha-AMS	Subretinal	MPD implant	VA ∼20/460–20/550; limited form vision.	(7, 10)
PRIMA	Subretinal	PV array (100 µm), wireless NIR	Native (no-zoom) VA ∼20/420; e-zoom ↑ recognition but ↓ FOV.	(11, 25, 35)
IMIE 256	Epiretinal	256-Ch HD array	↑ grating acuity & motion discrimination vs. 60-Ch devices.	(36)
BVA Gen-2	Suprachoroidal	44-Ch MEA	↑ functional vision & QoL over 2.7 y; acceptable safety; no device-related SAEs.	(28, 29)
Flexible arrays	Conformal	LM/CNT	Preclinical: ↓ impedance; ↑ charge-injection limit/durability.	(37–39)
Electronic photoreceptors	Subretinal	∼20 µm pixels	Preclinical: spatial resolution approaches ∼20/80 (rat).	(27, 43)
Te nanowire networks	Subretinal	TeNWNs (vis–NIR)	Preclinical: bias-free PV; robust responses in NHP.	(42)

VA/acuity-related endpoints are reported as described in the cited studies and may not be directly comparable across different testing paradigms.

BVA, bionic vision Australia; Ch, channel; CNT, carbon nanotube; e-zoom, electronic zoom; FOV, field of view; HD, high-density; LM, liquid metal; logMAR, logarithm of the minimum angle of resolution; MEA, microelectrode array; MPD, microphotodiode; NHP, non-human primate; NIR, near-infrared; PV, photovoltaic; SAE, serious adverse event; TeNWNs, tellurium nanowire networks; VA, visual acuity.

## 3 Optogenetics: gene transfer and opsin engineering

### 3.1 Classification of opsins and selection of target cells

The central premise of optogenetics-mediated vision restoration is to deliver genes encoding specific light-sensitive proteins (opsins) to surviving inner retinal neurons after retinal degeneration via viral vectors, thereby converting these cells into photosensitive “surrogate photoreceptors”. Currently, opsins used for vision restoration are broadly classified into microbial opsins [e.g., channelrhodopsins (ChRs)] and animal opsins; the two classes exhibit an inherent trade-off among light sensitivity, response kinetics, and signal-transduction mechanisms. Microbial opsins directly mediate transmembrane ion flux to drive rapid membrane depolarization and can support ultrafast (millisecond-scale) responses, but they generally have limited light sensitivity and require high-intensity illumination, raising concerns about potential phototoxicity (44–46). By contrast, animal opsins are derived from endogenous mammalian G protein–coupled receptors (GPCRs) and can achieve high light sensitivity through amplification via intracellular signaling cascades; however, their response latency is often on the order of seconds, which can markedly limit the perception of rapidly changing visual scenes (45, 47). Thus, opsin choice is inherently a compromise: gains in sensitivity or spectral range may come at the expense of temporal fidelity and motion perception.

Regarding target-cell selection, RGCs and bipolar cells are the two most widely investigated targets. Strategies targeting RGCs benefit from their relatively superficial anatomical location, enabling efficient transduction using engineered adeno-associated virus (AAV) vectors such as AAV2.7m8 (48). However, direct activation of RGCs bypasses the complex inner retinal signal-processing circuitry, and the resulting visual quality (e.g., spatial discriminability) may be limited (49). Accordingly, increasing attention has been directed toward more upstream bipolar cells—particularly ON-type (depolarizing) bipolar cells—to preserve endogenous parallel visual pathways to the greatest extent possible. For example, Opto-mGluR6, a chimeric protein that combines a melanopsin-based photosensitive domain with the intracellular domain of metabotropic glutamate receptor 6 (mGluR6), can be selectively targeted to ON bipolar cells and can mimic their physiological light response (17). Preclinical data indicate that optogenetic tools targeting bipolar cells can restore visual acuity and contrast sensitivity in animal models and elicit diverse receptive-field profiles in downstream RGCs, suggesting partial reconstitution of fine-grained retinal encoding (49). To achieve high-precision targeting, current studies are actively exploring cell-type-specific promoters [e.g., knock-in strategies based on clustered regularly interspaced short palindromic repeats (CRISPR)/CRISPR-associated (Cas) systems downstream of the endogenous glutamate metabotropic receptor 6 (GRM6) promoter], together with novel delivery platforms [e.g., exosome-associated AAV1 (Exo-AAV1)], to reduce ectopic expression in off-target cell types (50). Encouragingly, the spatial resolution of optogenetically reactivated retinas is determined by the receptive field size of transfected ganglion cells, which is confined to the soma and dendritic region rather than extending along axons (51). In late-stage disease, bipolar cells often exhibit adaptive remodeling, including dendritic retraction and altered protein expression profiles; these pathological changes may compromise transduction efficiency and therapeutic outcomes (52).

### 3.2 Milestones in clinical translation

Clinical translation of optogenetics for vision restoration has achieved notable milestones, but current leading paradigms remain coupled to dedicated external light-delivery hardware. A representative example is GS030, developed by GenSight Biologics, which uses the AAV2.7m8 vector to deliver the microbial opsin ChrimsonR to RGCs, together with engineered goggles that provide controlled, high-intensity patterned stimulation at 590 nm (irradiance >10^15^ photons/cm^2^/s) (48, 53). This reliance on high-intensity, goggle-based stimulation constitutes a fundamental physical constraint, particularly with respect to safety-limited irradiance (45), power consumption and battery life (15, 54)—factors that may limit the practical utility of optogenetic vision restoration in daily life. Importantly, the current clinical paradigm is not hardware-free: the gene therapy is coupled to a head-mounted camera–projector system and an external processing unit for real-time encoding and patterned stimulation.

**Figure 3:**
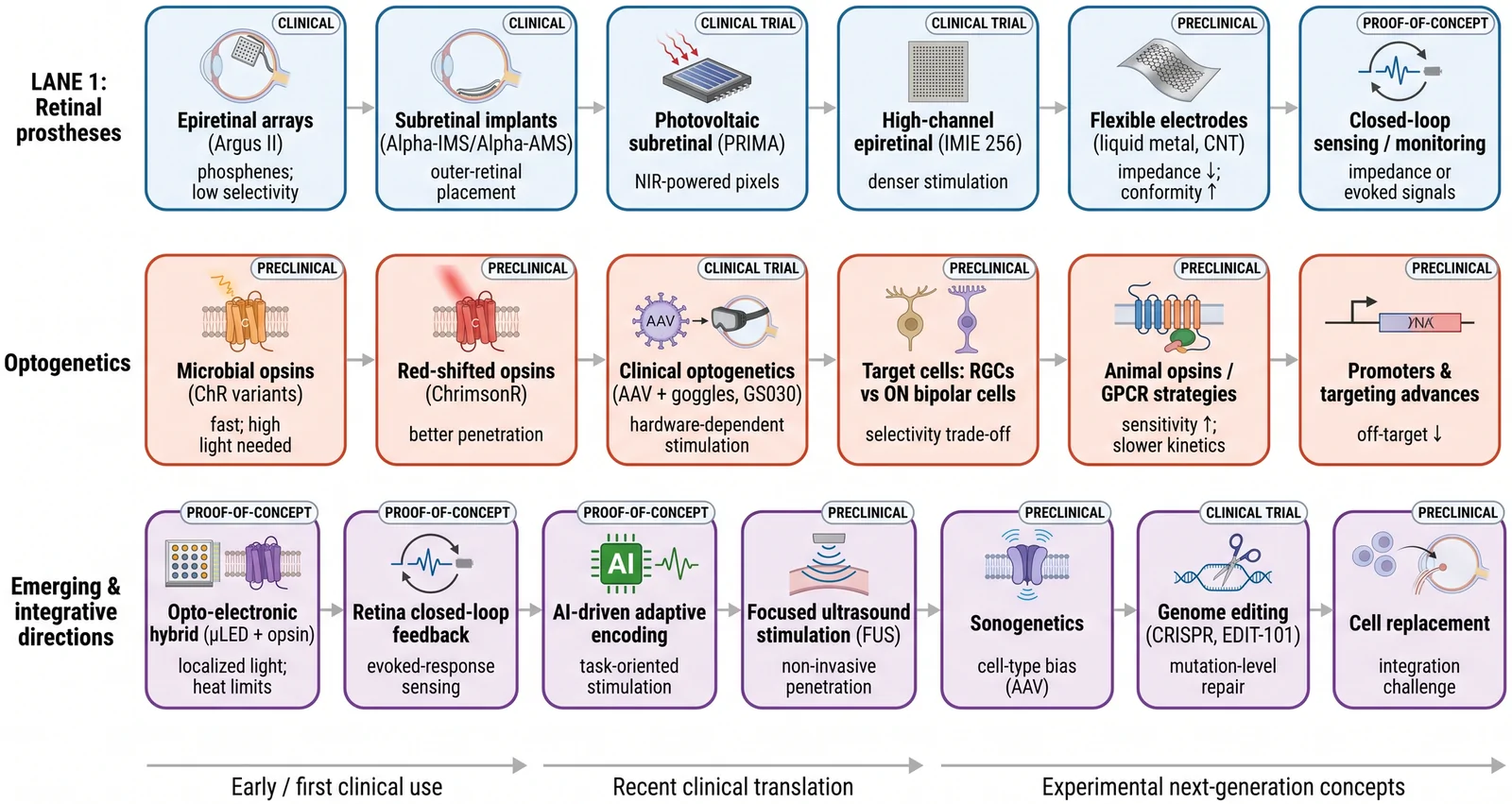
Key milestones across bionic vision strategies. Swimlane timeline summarizing representative milestones in retinal prostheses, optogenetics, and emerging/integrative directions. Each node is labeled by evidence maturity (e.g., clinical, clinical trial, preclinical, proof-of-concept) to emphasize that feasibility, safety, and usability constraints differ substantially across stages. The timeline highlights the transition from early electrode-array devices to higher-density, sensing-enabled interfaces; from opsin engineering to first-in-human optogenetic programs coupled to external light-delivery hardware; and toward experimental concepts such as µLED-opsin hybrids, retina-oriented closed-loop feedback, AI-assisted encoding, and acoustic neuromodulation. (Schematic generated with the assistance of Gemini 3.1 image editor.) AAV, adeno-associated virus; AI, artificial intelligence; CNT, carbon nanotube; CRISPR, clustered regularly interspaced short palindromic repeats; FUS, focused ultrasound; GPCR, G protein-coupled receptor; µLED, micro-light-emitting diode; NIR, near-infrared; ChR, channelrhodopsin; RGC, retinal ganglion cell.

Therefore, current clinical optogenetics should be evaluated as an integrated gene–device system rather than a stand-alone “gene-only” therapy. In its phase I/II clinical trial (NCT03326336), a report described a patient with advanced RP who, following a single intravitreal injection, regained the ability to perceive, localize, and count objects; electroencephalography (EEG) recordings showed corresponding activation patterns consistent with visual cortical engagement (53). Based on extrapolations from non-human primate models, the theoretical visual acuity (VA) achievable with this approach was estimated at approximately 20/249, supporting the feasibility of optogenetic vision restoration while highlighting that cohort-level efficacy and real-world usability still require further clinical validation (48). Importantly, early-phase outcomes do not yet define typical cohort-level benefit, and usability remains tightly coupled to the external stimulation chain. Milestones are annotated by evidence maturity to avoid conflating clinical validation with preclinical or proof-of-concept work. To contextualize these advances, key milestones across prosthetic, optogenetic, and emerging integrative approaches are summarized in Figure 3.

In addition, an open-label clinical study using a synthetic opsin (Synthopsin) has also reported encouraging results. In patients with blindness associated with ATP-binding cassette subfamily A member 4 (ABCA4) variants, a single injection of a broad-spectrum opsin—engineered through multiple mutations of a non-mammalian protein—was associated with improvements in visual acuity and shape discrimination, with preliminary evidence supporting the safety of a gene-only regimen (55). Strategically, the development of synthetic and broad-spectrum opsins can be viewed as an attempt to mitigate hardware constraints in current clinical optogenetics by improving effective photosensitivity and widening usable spectral bands, with the longer-term goal of lowering required irradiance and reducing dependence on high-intensity goggle-based stimulators, potentially enabling operation under more naturalistic illumination conditions in daily life, although this goal remains to be demonstrated in larger clinical datasets (55, 56). At present, the key translational question is whether such opsin engineering can meaningfully lower real-world irradiance requirements without sacrificing temporal resolution. Despite these advances and the absence of permanently implanted intraocular electrodes in current leading clinical programs, optogenetic vision restoration remains dependent on external high-intensity light sources, and clinically relevant constraints include opsin kinetics, phototoxicity-limited operating windows, vector-related intraocular immunogenicity, and variability introduced by non-uniform transgene expression (17, 45–49, 53, 55).

### 3.3 Bottlenecks: light intensity, spectral constraints, and dynamic range

For broader clinical deployment, next-generation optogenetic tools must address three major physical and physiological bottlenecks. First, high light intensity increases the risk of phototoxicity. For GS030, the light intensity required to activate ChrimsonR-transduced RGCs may approach or exceed commonly referenced retinal illumination safety limits (46, 48). Even for bipolar cell–targeted Opto-mGluR6, effective activation thresholds may leave a limited safety margin (46). Sustained high-intensity illumination can also drive opsin response saturation [e.g., with channelrhodopsin-2 (ChR2)] and may increase the risk of irreversible photochemical damage. To mitigate irradiance requirements, studies have reported upregulation of the voltage-gated potassium channel Kv1.3 in ON bipolar cells in degenerating retinas; selective inhibition of this channel through pharmacological approaches or gene silencing [e.g., knockdown of potassium voltage-gated channel subfamily A member 3 (KCNA3)] can enhance the amplitude and kinetics of Opto-mGluR6-mediated responses, thereby improving signal-to-noise performance at lower light intensities (52). In addition to photophysical constraints, clinical implementation must also account for biological safety, including the long-term tolerability of sustained opsin expression and the potential for AAV-related intraocular inflammation, which together shape the practical risk–benefit window for chronic light-driven stimulation (53, 57, 58). This means that “effective” stimulation is bounded not only by activation thresholds but also by cumulative exposure limits over years of use. Furthermore, transgene-related factors may shape long-term tolerability. Sustained high-level opsin expression can impose cellular burden (e.g., altered membrane conductance, metabolic stress, or unintended signaling interactions), potentially affecting neuronal health or response stability over years. Although clinically meaningful toxicity signals remain limited in current early-phase datasets, long-term surveillance is warranted, particularly when higher expression levels are required to compensate for light-sensitivity constraints.

Second, spectral penetration remains a constraint. Most natural opsins (e.g., ChR2) have peak sensitivity in the blue range (∼470 nm) (45). Blue light exhibits substantial scattering in ocular tissues, is readily absorbed by macular pigments, and carries a higher risk of photochemical damage than longer-wavelength light (46). Consequently, red-shifting opsin sensitivity has become an important engineering direction. Beyond ChrimsonR (activated at ∼600 nm), further development of deep red–shifted Opto-mGluR6 variants (e.g., ROM17, ROM18, and ROM19, with additional red shifts of 40, 70, and 126 nm, respectively) aims to improve tissue penetration while reducing phototoxicity under comparable activation efficacy (46).

Third, the dynamic range of current optogenetic systems is relatively narrow. The healthy human visual system can adapt over more than 10 log units of luminance via complex biochemical cascades involving rods and cones. In contrast, current opsins often exhibit steep input–output curves, with a dynamic range typically limited to ∼2–3 log units (59). As a result, patients may experience rapid saturation or signal loss when transitioning between lighting conditions (e.g., from a dim room to bright sunlight). Efforts to broaden dynamic range through opsin engineering are ongoing (59). In parallel, tighter coupling of photosensitive domains to endogenous G protein signaling pathways [e.g., the mGluR6–transient receptor potential melastatin 1 (TRPM1) cascade] is considered a promising approach to emulate physiological luminance adaptation and to expand the functional dynamic range of retinal information processing (26, 49). Notably, these bottlenecks are interdependent: strategies that increase light sensitivity or broaden dynamic range can impose trade-offs in kinetics, signal fidelity, and safety margins, and real-world performance can be further limited by heterogeneous transduction and non-uniform opsin expression across the retina (17, 45). Consequently, improving a single parameter (e.g., sensitivity) may not translate into better vision unless scattering, expression uniformity, and safety margins are co-optimized. Given the inherent trade-offs between light sensitivity and response speed, a detailed comparison of the biological mechanisms, kinetic properties, and translational status of major optogenetic tools is outlined in Table 2.

**Table 2:** Classification and engineering characteristics of optogenetic tools for vision restoration.

Opsin class	Mechanism of action	Response kinetics	Light sensitivity & dynamics	Representative examples	Target cells & status	Ref
Microbial opsins	Direct light-gated ion flux (membrane depolarization)	Fast (ms)	↓ sensitivity; DR ≈2–3 log units; high irradiance → phototoxicity risk; scattering/heterogeneous expression may reduce effective fidelity	ChR2 (∼470 nm); ChrimsonR (∼590–600 nm)	Mainly RGCs; ChR2 preclinical; ChrimsonR in clinical trial (GS030; goggles-coupled)	(45, 48, 53, 59)
Animal opsins (GPCR-based)	GPCR signaling cascades	Slow (s; often seconds)	↑ sensitivity (signal amplification); potential for more physiological luminance processing; practical limits include remodeling/transduction variability	Opto-mGluR6; red-shifted Opto-mGluR6 variants (ROM17/18/19)	ON BCs; preclinical	(17, 46, 47, 49, 58)
Synthetic opsins	Engineered opsins (multi-mutation design)	Tunable (program-dependent)	Broad spectrum; aims to ↑ effective photosensitivity and ↓ reliance on high-intensity hardware; real-world irradiance needs validation	Synthopsin (broad-spectrum)	Retinal cells; clinical (open-label; ABCA4-associated blindness); potential “gene-only” regimen	(55)

Practical performance can be constrained by safety-limited irradiance, optical scattering, limited DR, AAV-related inflammation risk, and heterogeneous transduction.

AAV, adeno-associated virus; ABCA4, ATP-binding cassette subfamily A member 4; BC, bipolar cell; ChR2, channelrhodopsin-2; DR, dynamic range; GPCR, G protein–coupled receptor; ms, millisecond; nm, nanometer; ON, ON-pathway (depolarizing); RGC, retinal ganglion cell; s, second.

## 4 Core comparisons: resolution, adaptation, and biosafety

### 4.1 Spatial resolution and visual quality

In bionic vision technologies, spatial resolution and the quality of reconstructed vision are key benchmarks for evaluating intervention performance. For retinal prostheses, the upper limit of functional resolution is constrained by the physical pixel density of the microelectrode array or photovoltaic chip, as well as by stimulation spread and non-selective pathway recruitment. Across early-generation clinical implants, reported acuity-related endpoints and task-based measures generally remain well below the legal blindness threshold (20/200), although heterogeneous testing paradigms and endpoints complicate direct cross-study comparison. For example, in epiretinal Argus II users, reported acuity-related endpoints in some paradigms correspond to approximately 20/1260 (10). Because acuity endpoints differ across studies (e.g., grating-based measures vs. letter-based acuity), direct numerical comparisons should be interpreted conservatively. Subretinal prostheses (e.g., Alpha-AMS) have typically been reported to range from 20/460 to 20/550 in selected cohorts (10).

Notably, newer high-density photovoltaic prostheses have shown the potential to partially overcome this bottleneck. Clinical data suggest that PRIMA recipients can achieve a mean native (no-zoom) acuity of approximately 20/420, broadly consistent with a 100 µm physical pixel size, while earlier follow-up reports have described values around 20/500 in some cohorts (25, 35). Importantly, electronic zoom can improve character recognition and related task performance, but at the cost of a restricted effective field of view, which should be explicitly considered when comparing platforms (25, 35). Preclinical studies in rat models, together with device-level modeling, further suggest that reducing pixel size to ∼20 µm may enable acuity approaching ∼20/80 under idealized conditions; however, such performance has yet to be validated for durable, cohort-level benefit in humans (27, 43).

Optogenetics is often discussed as offering potentially higher spatial selectivity because optical stimulation can, in principle, be patterned at cellular scales (26, 51). In practice (with evidence derived mainly from preclinical studies), effective resolution can be attenuated by light scattering in retinal tissue, safety-limited irradiance, and heterogeneous transduction due to non-uniform opsin expression, which together reduce spatial fidelity and perceptual consistency (26, 54, 60, 61). Accordingly, “single-cell theoretical resolution” is not an appropriate proxy for expected clinical vision quality under safety-limited stimulation. Overall, retinal prostheses currently have an advantage in near-term clinical accessibility—stimulation can be delivered immediately after device activation—whereas optogenetics has longer-term potential for higher-fidelity encoding if cell-type–biased reactivation and safer, lower-irradiance operation can be achieved. At present, both approaches primarily support limited functional vision in selected patient populations, and a substantial technological gap remains before high-quality, naturalistic vision can be reliably achieved (62).

### 4.2 Neural adaptation and plasticity

Neural adaptation and central plasticity are critical determinants of whether “the brain can effectively decode bionic” inputs. In retinal prosthesis users, prosthesis-evoked percepts can differ fundamentally from the spatiotemporal coding of the intact retina, and recipients may require a prolonged process of cortical adaptation and task-specific training. For example, Argus II users commonly describe their visual input as discrete, flickering light spots. Given the limited field of view of a head-mounted camera, users must scan and integrate scenes through continuous head movements (63). This is followed by a structured rehabilitation process spanning weeks to months, during which patients learn to interpret the discontinuous phosphene patterns as meaningful visual contours for daily tasks (8, 10, 11). Importantly, this decoding burden is not solely a “learning” issue but is driven by physiological incongruence at the stimulation level: conventional extracellular electrical stimulation can non-selectively recruit overlapping ON (depolarizing) and OFF (hyperpolarizing) pathways and may also activate RGC axons in the nerve fiber layer, resulting in spatially distorted and temporally ambiguous signals delivered to the brain (11, 12). As a result, the cortex is tasked with interpreting inputs that are, to some extent, physiologically incoherent relative to natural retinal encoding, which can slow adaptation and limit performance in dynamic visual scenes. This framing emphasizes that adaptation is constrained by the coherence of the retinal code delivered to the cortex, not merely by user motivation or training duration. Notably, the adaptation burden and the immediacy of usable form vision can vary across platforms and patient populations and should not be generalized to all retinal prostheses (10, 34).

Optogenetics may offer intrinsic advantages for neural adaptation in principle; however, these advantages are supported mainly by preclinical studies, and robust human evidence remains limited. By selectively targeting retinal cell populations (e.g., ON-type bipolar cells), optogenetics can reactivate endogenous parallel processing mechanisms within the retina and may provide the brain with signals that more closely reflect the structure of physiological pathways than direct RGC activation alone (17, 49). However, evidence supporting superior central adaptation in humans remains limited, as many mechanistic insights are derived primarily from rodent and non-human primate studies. In addition, real-world performance can be constrained by disease-stage remodeling, heterogeneous transduction, and the need for dedicated external light-delivery systems in current clinical paradigms, all of which can affect the stability and interpretability of the retinal output (16, 49). Moving forward, elucidating how the brain decodes non-physiological bionic inputs and optimizing stimulation and rehabilitation paradigms to reduce the cortical decoding burden remain key challenges for meaningful functional vision restoration (4, 8, 34, 64).

### 4.3 Safety profile

Safety is fundamental to assessing the long-term feasibility of these two emerging technologies. Their risk profiles differ substantially: the safety of retinal prostheses is mainly determined by invasive surgery and chronic foreign-body responses to implanted hardware, whereas optogenetics is influenced by the biological effects of gene-delivery vectors and sustained light exposure at or near safety limits.

For retinal prostheses, unavoidable surgical trauma may lead to acute complications such as retinal detachment and endophthalmitis. In addition, chronic fibrosis induced by foreign-body responses can reduce electrode performance and may cause mechanical traction on the retina. Some systematic reviews have suggested that epiretinal implantation (including Argus II) may be associated with a higher overall adverse-event burden than implants at other anatomical sites, although cross-study comparisons are limited by heterogeneous reporting and follow-up (10). A systematic review and meta-analysis of harms associated with retinal implantation of stimulating electrode arrays for outer-retinal degeneration synthesized 59 peer-reviewed articles covering 11 devices and emphasized substantial heterogeneity in harms definitions and reporting quality (McMaster scores 3–12/15), limiting robust incidence estimation and cross-device comparisons (33). At the review level, the authors compiled a comprehensive catalogue of serious and non-serious adverse events and identified several complication types that were repeatedly reported across the literature. In their summary of commonly reported harms, conjunctival dehiscence/erosion was the most frequently reported event (58 total; 16 serious), followed by implant displacement (44 total; 3 serious) and hypotony (40 total; 12 serious); retinal detachment (all types) was likewise a prominent recurrent harm (14 total; 9 serious) (33). Notably, the review highlighted that the reported incidence of these complications varied widely across individual studies, underscoring strong dependence on device- and procedure-specific factors. To illustrate this profound heterogeneity, the authors extracted incidence ranges of approximately 7.3% to 56% for conjunctival erosion/dehiscence, 4% to 23% for hypotony events, 6% to 60% for retinal detachments (with retinoschisis reaching 45% in one analyzed cohort), and 10% to 25% for implant displacements across different platforms (33).

Beyond cataloguing individual harms, the systematic review by Hallum and Cloherty (33) provided several methodological insights that merit explicit consideration. First, the authors demonstrated that serious adverse events follow a log-uniform temporal distribution, with a higher density early post-implantation and a long-tail accrual over follow-up. This finding challenges the common assumption that safety can be adequately assessed within short observation windows, and carries direct implications for clinical trial design and post-market surveillance. Second, the review explicitly noted that rigorous comparison of safety profiles across different implant locations—epiretinal, subretinal, or suprachoroidal—is not supported by the current evidence base, as most data are derived from single-arm studies without standardized ascertainment of harms. This observation underscores that claims of superior safety for any particular surgical approach should be interpreted with caution until comparative data become available. The review further noted that standardized quantitative assessments of baseline vision were often lacking, and the psychometric methods used to evaluate prosthesis-mediated vision were subject to methodological limitations and inconsistent reporting across studies (65–67).

Taken together, these quantitative findings reinforce our central premise: the safety of retinal prostheses is not only an acute surgical concern but also an enduring biointerface challenge, in which long-term mechanical stability and tissue tolerance are tightly coupled to the durability and fidelity of prosthetic visual encoding. Moreover, metallic implants can raise electromagnetic-compatibility concerns, potentially restricting access to certain magnetic resonance imaging (MRI) examinations. Some devices (e.g., Alpha-IMS) may also limit upward and temporal eye movements due to extraocular cable routing, although this typically does not severely compromise monocular vision or routine device utility (68).

**Figure 4:**
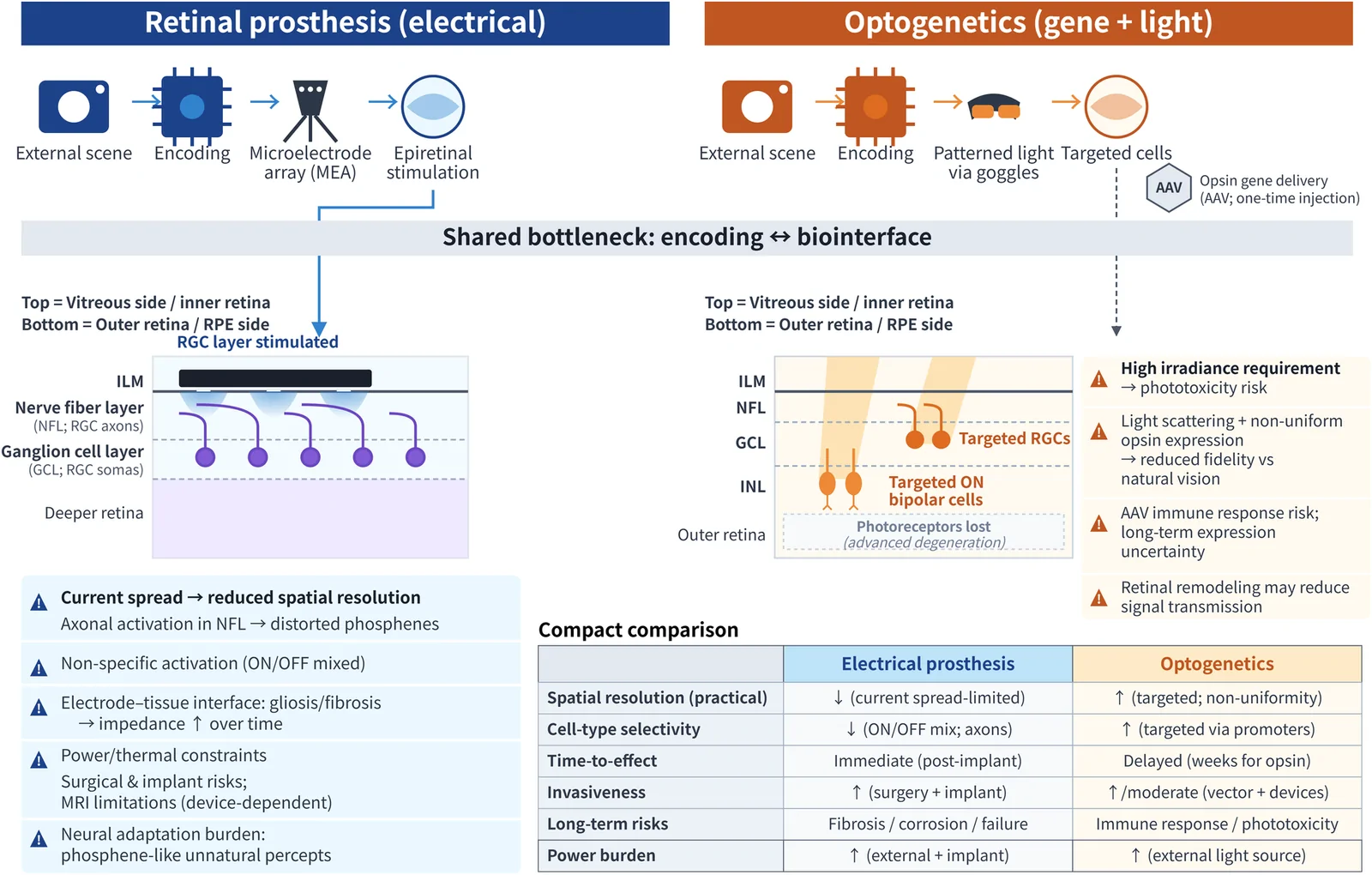
Encoding–biointerface bottleneck comparison: electrical retinal prostheses vs. optogenetics. Side-by-side schematic spanning external scene capture/encoding, stimulation delivery, and an anatomically accurate retinal interface context for electrical prostheses and optogenetics. The central band highlights the shared “encoding–biointerface” bottleneck. Left: In an epiretinal electrical prosthesis (illustrative example), a microelectrode array (MEA) is positioned on the vitreous side overlying the inner limiting membrane (ILM)/nerve fiber layer (NFL). Practical selectivity is limited by current spread and non-selective recruitment of NFL axons, contributing to spatially distorted percepts and a high neural-adaptation burden. Right: In optogenetic vision restoration, the gene–device chain (AAV-mediated opsin delivery to inner-retinal target cells, e.g., retinal ganglion cells and/or ON-pathway bipolar cells, coupled to external patterned-light hardware in current clinical paradigms) is constrained by safety-limited irradiance (phototoxicity risk), optical scattering, heterogeneous expression (reduced spatial fidelity), and vector-related immune responses and long-term expression uncertainties. The bottom panel provides a compact cross-modality summary of trade-offs in resolution, cell-type selectivity, invasiveness, time-to-effect, and long-term risks. (Schematic generated with the assistance of Gemini 3.1 image editor.) AAV, adeno-associated virus; GCL, ganglion cell layer; ILM, inner limiting membrane; INL, inner nuclear layer; MEA, microelectrode array; NFL, nerve fiber layer; ON, depolarizing; RGC, retinal ganglion cell; RPE, retinal pigment epithelium.

In contrast, safety concerns for optogenetics reflect combined biochemical and photophysical risks. On the one hand, AAV vectors used for gene transfer have a very low but nonzero long-term risk of genomic integration, and AAV capsids can trigger host immune responses, potentially leading to delayed intraocular inflammation (69). On the other hand, the optical power required to activate low-sensitivity opsins—particularly under chronic or repeated use—may increase the risk of retinal phototoxicity and secondary injury to already fragile tissue (15). In summary, prosthesis development requires continued advances in long-term material biocompatibility and interface stability, whereas optogenetics warrants rigorous long-term pharmacovigilance for both gene delivery and cumulative light exposure. This bottleneck is not purely hardware- or gene-limited; it reflects the joint constraints of stimulation selectivity, tissue safety margins, and the brain's decoding and adaptation. To visualize how constraints accumulate from external scene encoding to the retinal interface and downstream decoding demands, we summarize both systems side by side. Across spatial resolution, neural adaptation, and safety, both approaches converge on a shared “encoding–biointerface” bottleneck that critically limits clinical fidelity and usability (Figure 4).

For both approaches, large-scale, long-term real-world follow-up is necessary to more comprehensively characterize safety profiles and to inform appropriate risk-management strategies. Such registries are also essential for capturing late-onset complications and for defining pragmatic, device-specific risk-management thresholds.

## 5 Future directions: hybrid strategies and intelligent systems

### 5.1 Optogenetic–electronic hybrid interfaces

Optogenetic–electronic hybrid interfaces have been proposed as an experimental frontier in bionic vision, with the central concept of combining the cell-type–biased targeting of optogenetics with electrical stimulation delivered through implantable electrode arrays, which can reduce optical thresholds and improve response reliability (12). Meanwhile, the development of implantable micro-light-emitting diode (µLED) arrays offers a complementary approach for delivering localized optical stimulation in preclinical settings (21). In principle, implantable high-density µLED arrays could provide highly localized optical stimulation, and proof-of-concept studies have demonstrated their ability to evoke retinal ganglion cell responses in *ex vivo* retinal preparations (54, 70, 71). Specifically, implantable µLED arrays can independently control individual micro-pixels via serial addressing with row- and pixel-based random updating, with a pixel pitch of 80 × 80 µm (containing 20 µm LED emitters), and can illuminate target cells with a measured median peak irradiance of 1.35 mW/mm^2^ (54). Such localized illumination has been shown to enable effective activation of opsins in proof-of-concept retinal preparations (54, 71, 72). Because optical stimulation is spatially confined and activation thresholds can be more precisely controlled, this approach may also reduce overall power consumption and extend the operating time of external wireless wearable components (reported to be ∼2 h of continuous use) (70).

Nevertheless, this hybrid strategy remains preclinical, and several translational barriers are particularly stringent for intraocular implants. Beyond the need for highly precise and minimally invasive implantation, key challenges include thermal management of high-density µLED arrays (72, 73), long-term encapsulation stability and biocompatibility in the ocular environment (11, 37), and precise spatial alignment between the stimulating electrodes and target retinal neurons to minimize electrode-retina distance (11, 37, 71). These constraints underscore that optoelectronic hybrids should currently be interpreted as engineering proof of concept rather than near-term clinical solutions. Until chronic thermal safety and stable long-term packaging are demonstrated *in vivo*, clinical timelines should be considered uncertain.

### 5.2 Closed-loop adaptive encoding

To move from “detecting light” to “interpreting scenes”, closed-loop strategies have been proposed for retinal prostheses and are facilitated by measuring electrically evoked retinal responses for feedback control and model-based optimization (74). In parallel, deep-learning–based, task-oriented encoding frameworks have been demonstrated in proof-of-concept cortical visual prosthesis pipelines to extract task-relevant scene features and generate stimulation patterns (75).

However, it is essential to distinguish evidence across anatomical targets and device classes. For example, a deep convolutional neural network–based neural interface has been developed to translate visual inputs into stimulation sequences tailored for cortical prostheses, demonstrating the conceptual promise of task-oriented encoding and feedback-driven optimization in a non-retinal setting (75). This cortical literature is informative as a proof-of-concept framework, but it does not directly establish the feasibility of intraocular devices, in which constraints on heat dissipation, implant volume, long-term hermetic packaging, and corrosion resistance are substantially more stringent (11, 21, 73). As a result, algorithmic “promise” must be judged against ocular hardware constraints, not solely against decoding performance in benchtop demonstrations. Retinal-oriented encoding and closed-loop strategies have begun to emerge but remain at an early experimental stage, and their real-world benefits and safety trade-offs require further validation (74). Importantly, many retina-oriented demonstrations currently focus on measuring evoked responses for feedback control, which is a necessary step but not yet equivalent to clinically robust scene-level closed-loop vision. Thus, today's “closed loop” is often parameter-level control rather than perception-level scene optimization.

**Figure 5:**
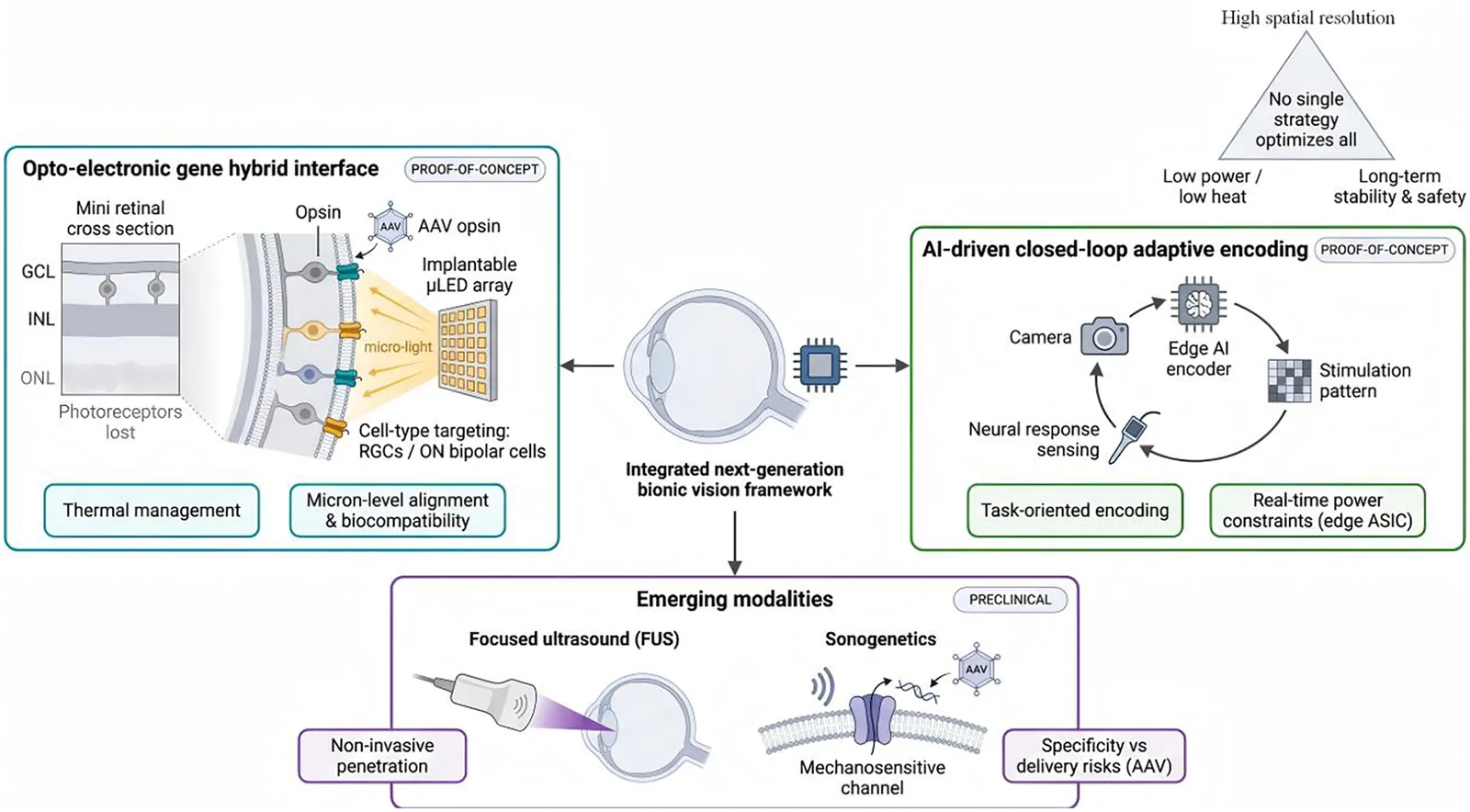
Integrated next-generation framework and trade-offs. Systems framework integrating three experimental pillars for next-generation bionic vision: (i) opto-electronic gene hybrid interfaces that combine AAV-mediated opsin expression in surviving inner retinal cells (e.g., RGCs or ON bipolar cells) with implantable µLED arrays for localized stimulation; (ii) AI-driven closed-loop adaptive encoding that links sensing, edge encoding, stimulation pattern generation, and feedback based on neural response measurements; and (iii) emerging modalities such as focused ultrasound (FUS) and sonogenetics that trade implantation invasiveness for penetration depth and genetic specificity. The trade-off triangle highlights the intrinsic tension among high spatial resolution, low power/low heat, and long-term stability/safety, underscoring that no single strategy optimizes all constraints simultaneously. (Schematic generated with the assistance of Gemini 3.1 image editor.) AAV, adeno-associated virus; AI, artificial intelligence; ASIC, application-specific integrated circuit; FUS, focused ultrasound; µLED, micro-light-emitting diode; RGC, retinal ganglion cell.

From an engineering perspective, practical implementation faces a fundamental trade-off between computational demand and energy consumption: real-time artificial intelligence (AI) inference typically requires low-power, high-performance application-specific integrated circuits (ASICs) to meet strict thermal and power constraints, particularly for intraocular systems (76, 77). In addition, aligning externally generated encoding with endogenous neural processing to avoid perceptual conflicts or disorganized percepts, and maintaining algorithmic robustness under extreme lighting conditions remain central challenges for future software development (11). Given that preclinical or early-stage proof-of-concept data predominantly support these evolving strategies, an integrated framework is useful for separating feasibility from speculation and for making ocular constraints explicit. Future progress will likely depend on careful co-design of stimulation hardware, encoding algorithms, and patient-specific calibration and training, while explicitly balancing resolution, power and thermal limits, and long-term stability and safety. A systems-level view of these trade-offs is outlined in Figure 5.

### 5.3 Targeted genome editing and cell replacement

For end-stage disease with near-complete photoreceptor loss, targeted genome editing and cell replacement therapies offer potential routes to reconstruct visual circuits at a more fundamental level, but their translational scope differs from that of bionic interfaces and is often disease- and stage-dependent. In genome editing, CRISPR–Cas9 (CRISPR-associated protein 9)-based approaches aim to correct pathogenic variants underlying inherited retinal degeneration at the molecular level. A representative example is EDIT-101, an *in vivo* editing therapy targeting CEP290 mutations; this program has advanced to clinical testing and has provided initial evidence supporting the feasibility of intraocular genome editing (57). However, delivery efficiency, durability of benefit, and potential off-target effects require careful evaluation in larger cohorts with extended follow-up, and these approaches are not broadly applicable to all causes of advanced retinal degeneration (57).

For cell replacement, transplantation of highly purified photoreceptor precursor cells derived from pluripotent stem cells represents another major strategy. Current bottlenecks include controlling immune rejection in allogeneic settings and promoting functional synaptic integration between donor cells and the host inner retina (e.g., bipolar cells) (11, 62). In addition, a broader concept of functional cell replacement—namely, targeted delivery of genes such as Opto-mGluR6 to surviving ON-type bipolar cells—has been reported to reshape ON/OFF parallel pathways without requiring high-intensity light in the rd1 (retinal degeneration 1) mouse model, providing an alternative framework for retinal reprogramming (58). These disease-modifying approaches address different clinical questions than bionic interfaces do, and their timelines and applicability are mutation- and stage-specific. Looking ahead, integrating *ex vivo* gene correction of autologous stem cells with directed differentiation and transplantation may help reduce immune barriers and improve graft–host integration, although substantial technical challenges remain.

### 5.4 Ethics and accessibility

Rapid advances in bionic vision and gene-based neuromodulation inevitably raise important considerations in neuroethics and health economics. Ethically, highly integrated closed-loop brain–computer and brain–eye interfaces may affect embodied cognition and can introduce psychological and social challenges during adaptation to “hybrid” sensory function. Moreover, because these interventions—including viral vector–based gene therapies and potentially irreversible neural implants—remain in their early stages for many indications, ensuring valid informed consent under conditions of substantial uncertainty, alongside rigorous trial oversight, transparent outcome reporting, and adherence to pre-registered endpoints, is fundamental to mitigating the risk of premature over-commercialization before adequate safety and efficacy evidence is established (11, 66, 78).

From an accessibility perspective, high research, manufacturing, and implementation costs remain major barriers to broad global adoption. In addition to direct device and procedural costs, real-world deployment depends on long-term service infrastructure, software and hardware maintenance, and sustained commercial and clinical support, which have been limiting factors for some first-generation platforms (8). Advanced optogenetic or genome-editing programs such as GS030 typically require customized viral vector production and dedicated hardware, which can be costly; moreover, some gene therapies may show waning efficacy and face repeat-dosing challenges due to neutralizing antibodies, further increasing the long-term financial burden (50, 53, 55, 57). To address these challenges, decentralized clinical registries and long-term monitoring using real-world data (RWD) can help evaluate safety, durability, and health-economic value, thereby informing reimbursement models and improving access for a broader population of individuals with blindness.

## 6 Emerging technologies: ultrasound stimulation and sonogenetics

### 6.1 Principles and potential advantages of ultrasound stimulation

As an emerging non-invasive neuromodulation modality, focused ultrasound (FUS) has introduced a third physical intervention pathway for vision restoration. Its proposed mechanism leverages the relatively high tissue penetration of ultrasound to deliver acoustic energy through the ocular refractive media and elicit action potentials in retinal neurons via mechanical effects (e.g., acoustic radiation force) (20). Compared with conventional electronic retinal prostheses, a key potential advantage of ultrasound-based stimulation is the absence of implanted intraocular hardware, which may reduce risks associated with complex ocular surgery, foreign-body reactions, and long-term degradation of the tissue–electrode interface. Compared with optogenetics, early ultrasound approaches do not require viral vector–mediated gene transfer, thereby avoiding gene-therapy-related risks in principle (20, 50, 53).

In terms of stimulation precision, preclinical rat studies have reported spatial resolutions of approximately 250 µm and temporal resolutions of 5 Hz, which may be sufficient to encode coarse spatiotemporal patterns but remain far below the spatial resolution required for vision approaching the legal blindness threshold (79). Therefore, current non-invasive ultrasound approaches are better viewed as complementary modalities for coarse stimulation rather than stand-alone routes to high-acuity vision. Although diagnostic ultrasound has a longstanding clinical safety record, transocular neuromodulation for repeated or chronic use requires dedicated safety evaluation, including ocular-interface heating and mechanical bioeffects under neuromodulatory exposure conditions.

### 6.2 Preclinical evidence and engineering challenges

The feasibility of FUS for vision restoration has been preliminarily evaluated in multiple animal models. In rats with intact visual pathways, non-invasive ultrasound stimulation can trigger retinal neuronal firing and elicit responses in higher visual centers, such as the superior colliculus and the primary visual cortex (V1) (79). Importantly, in the Royal College of Surgeons (RCS) rat model of severe photoreceptor degeneration, FUS has been reported to activate surviving inner retinal networks and elicit responses measurable in the visual cortex, supporting its potential applicability to degenerative retinal disease at a proof-of-concept level (79). In addition, by customizing ultrasound phased arrays or acoustic lenses (e.g., a 4.4 MHz spiral transducer), investigators have generated static acoustic focal patterns resembling specific letters *in vivo*, suggesting that patterned stimulation may be achievable under controlled experimental conditions (79).

Nevertheless, translating ultrasound retinal stimulation from proof of concept to clinical application poses substantial engineering challenges. First, miniaturization and wearable integration remain major hurdles: current transducers for transocular ultrasound are typically bulky, and practical systems will likely require compact, low-power phased arrays compatible with a smart-glasses–like form factor (20). Second, it remains necessary to balance ultrasound frequency (to improve spatial resolution) against penetration depth, while minimizing standing-wave formation and excessive heating at ocular interfaces such as the lens; these issues require further acoustic modeling and *in vivo* safety validation.

**Figure 6:**
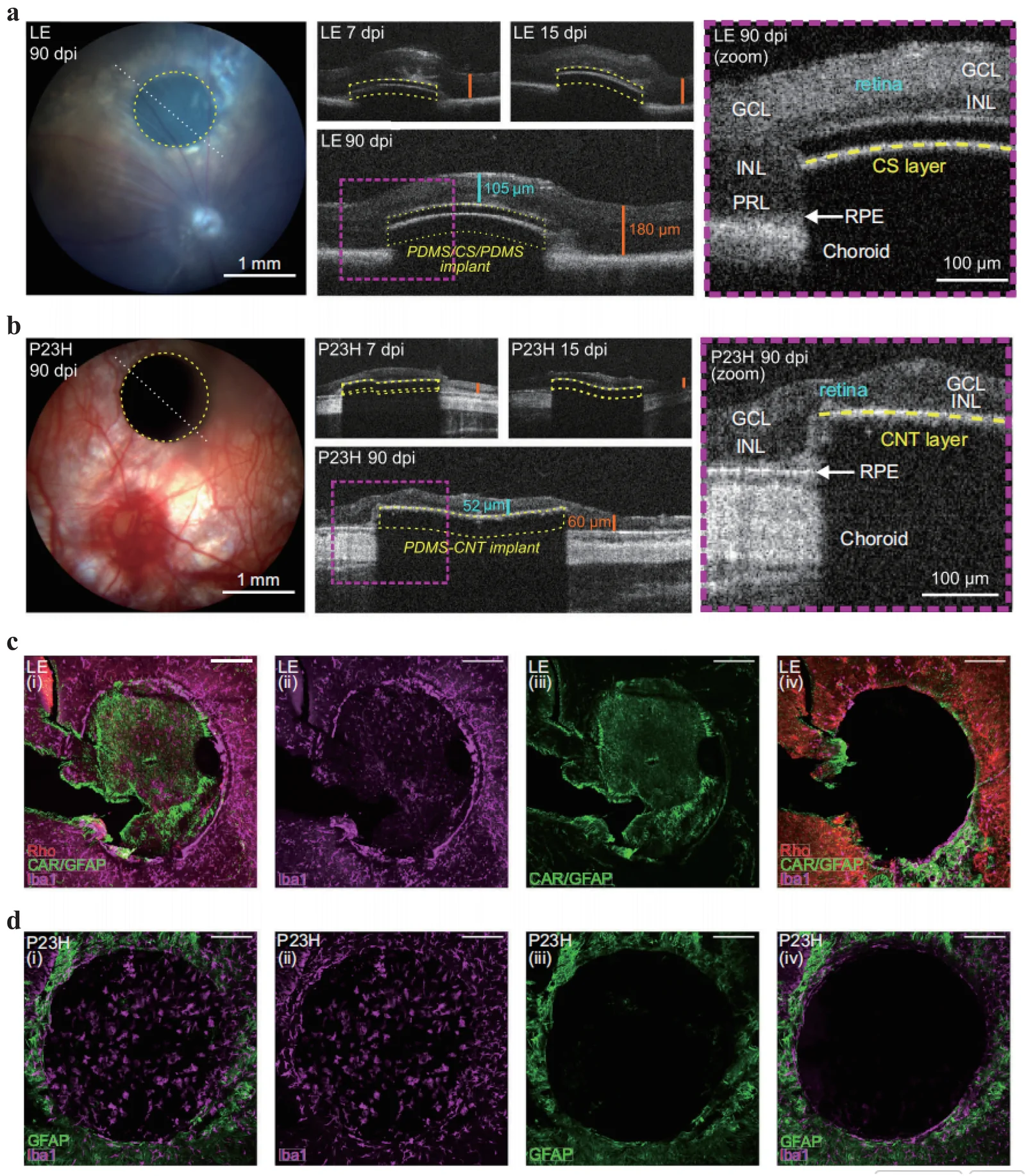
*In vivo* localization and biocompatibility assessment of a flexible photoacoustic subretinal film implant (fundus, OCT, and immunolabeling) (80). **(a)** Representative fundus photograph (left) and longitudinal OCT B-scans (middle and right) in a Long Evans rat showing a subretinal PDMS/CS/PDMS implant (yellow dotted outline) at multiple time points post-implantation; OCT was acquired along the indicated scan line and illustrates layer relationships (e.g., GCL, INL, PRL, RPE) and structural changes above the implant over time. **(b)** Same imaging workflow in a P23H retinal degeneration rat. **(c,d)** Immunolabeling of retinal tissue in implanted regions and surrounding zones, including photoreceptor markers (Rho, CAR) and glial/microglial markers (GFAP, Iba1), to evaluate local tissue responses under the reported conditions. Together, these multimodal readouts provide anatomical and histological evidence relevant to longitudinal implant monitoring and translational feasibility. [Reprinted from (80), under CC BY-NC-ND 4.0.] CAR, cone arrestin; CNT, carbon nanotube; CS, carbon sheet; dpi, days post-implantation; GFAP, glial fibrillary acidic protein; GCL, ganglion cell layer; Iba1, ionized calcium-binding adapter molecule 1; INL, inner nuclear layer; LE, Long Evans; OCT, optical coherence tomography; PDMS, polydimethylsiloxane; PRL, photoreceptor layer; Rho, rhodopsin; RPE, retinal pigment epithelium.

To address the coarse spatial resolution of non-invasive FUS, emerging implant-assisted concepts, such as implantable photoacoustic films, have been proposed to enhance spatially structured retinal responses while retaining external acoustic drive, but these remain preclinical and require validation for long-term safety and durability (80). Beyond spatial precision, implant-assisted photoacoustic approaches must demonstrate stable subretinal positioning and acceptable tissue responses during longitudinal follow-up. *In vivo* fundus photography and OCT provide anatomical evidence for implant localization and retinal layer relationships, while immunolabeling of implanted regions supports evaluation of local glial/microglial responses under the reported conditions. Representative imaging- and histology-based evidence for a flexible photoacoustic subretinal film implant is shown in Figure 6.

### 6.3 Sonogenetics: combining physical penetration with molecular specificity

To address the limited cellular selectivity of ultrasound stimulation alone, sonogenetics has emerged as a concept that combines ultrasound's penetration with genetically enabled, cell-type–biased sensitivity. The central approach is to use viral vectors to drive ectopic expression of exogenous mechanosensitive ion channels in target retinal neurons (e.g., selected RGC subtypes or bipolar cells), such as the mechanosensitive channel of large conductance (MscL) and its G22S mutant, or transient receptor potential vanilloid 1 (TRPV1) (20, 21). Under relatively small mechanical stresses induced by FUS, engineered channels may open more readily than endogenous mechanosensitive pathways, thereby increasing cation influx and potentially improving the sensitivity and signal-to-noise ratio of ultrasound-evoked neuronal responses in proof-of-concept settings (20, 21, 81).

Sonogenetics may help mitigate optogenetic limitations related to optical scattering and phototoxicity. However, important limitations should be acknowledged. Although stimulation is non-invasive with respect to implantation, sonogenetic preparation typically requires intraocular delivery of AAV vectors and therefore carries gene-therapy-related risks, including immunogenicity and uncertainties regarding long-term expression stability (20, 55, 57). At present, sonogenetic visual prostheses remain in their early stages, and key priorities include establishing safety and durability in large-animal models, defining safe acoustic dose ranges for chronic use, and developing miniaturized ultrasound emitters suitable for daily-life deployment (20). To contextualize conventional and emerging bionic vision strategies, Table 3 presents a cross-modality comparison of stimulation mechanisms, resolution limits, and primary safety bottlenecks across electrical, optical, and acoustic neuromodulation approaches.

**Table 3:** Comparative summary of bionic vision modalities: electrical, optical, and acoustic neuromodulation.

Parameter/feature	Electronic retinal prostheses	Optogenetics (gene therapy)	Focused ultrasound (FUS)/sonogenetics
Stimulation mechanism	Extracellular electrical pulse trains (8).	Light-activated ion channels or GPCR cascades (45, 47).	Mechanical acoustic effects (FUS) and/or genetically enabled mechanosensitive channels (sonogenetics) (20, 21, 81).
Translational status/evidence maturity	Clinical (early-generation) and clinical trials/updates for newer systems (8, 25, 28, 29, 35, 36).	Clinical trial (hardware-coupled paradigms) and preclinical (53, 55).	Preclinical/proof-of-concept (20, 79–81).
Hardware dependency	Permanent intraocular array plus external camera/processor/transmission; device-specific external components (e.g., NIR projection for PV systems) (8, 11, 35).	No intraocular electrode array in current leading paradigms; requires dedicated external high-intensity patterned-light hardware (e.g., goggles) (53).	External/wearable ultrasound transducer (arrays/lenses); sonogenetics additionally requires vector delivery (20, 79).
Spatial resolution limit	Limited by pixel density and current spread (clinical PV pixel pitch ∼100 µm; preclinical small-pixel designs ∼20 µm) (25, 27, 35, 43).	Theoretical high selectivity; practical limits include scattering and heterogeneous expression/transduction (26, 51).	∼250 µm (preclinical FUS); may be enhanced via implant-assisted photoacoustic films (still preclinical) (79, 80).
Cellular selectivity	Low; ON/OFF mixing and axonal activation can occur (11, 12).	Higher with targeted expression (e.g., ON BCs), but non-uniformity limits practical fidelity (17, 49, 50).	Low (FUS alone) to higher (sonogenetics with cell-type targeting) (20, 21, 81).
Primary safety bottlenecks	Surgical trauma; chronic fibrosis/gliosis; long-term device-related complications/harms (10, 33).	Safety-limited irradiance (phototoxicity risk); AAV-related inflammation/immune response; long-term expression uncertainty (46, 53, 69).	Ocular-interface heating and mechanical bioeffects; safe dose limits; standing waves/targeting constraints (20, 79).
Cortical neural adaptation	Often high decoding/training burden; platform- and patient-dependent (34, 64).	Potentially more physiological with upstream targeting (e.g., bipolar cells), but robust human evidence remains limited (17, 49, 53).	Proof-of-concept responses reported in higher visual centers (e.g., V1/superior colliculus) in animal models (79).

Statements are summarized from the cited literature and may not be directly comparable across studies due to differences in testing paradigms, disease stage, and device configurations. Evidence maturity reflects the general maturity of published evidence rather than a uniform regulatory status across all subtypes.

AAV, adeno-associated virus; BC, bipolar cell; FUS, focused ultrasound; GPCR, G protein–coupled receptor; µm, micrometer; NIR, near-infrared; PV, photovoltaic; V1, primary visual cortex.

## 7 Conclusion

Retinal prostheses and optogenetics both show clinical feasibility for advanced retinal degeneration but remain constrained by the encoding–biointerface bottleneck: electrical stimulation is limited by current spread, mixed pathway/axon recruitment, and long-term interface reactions that increase cortical decoding burden, whereas optogenetic strategies—despite cell-type–biased reactivation—are currently hardware-coupled and restricted by safety-limited irradiance, optical scattering, heterogeneous transduction, limited dynamic range, and AAV-related inflammatory risk. Consequently, meaningful functional vision will likely require multidisciplinary integration rather than a single replacement technology, combining improved biointerfaces and materials (including broadband subretinal nanomaterial-based photovoltaic nanoprostheses), retina-oriented adaptive encoding and calibration, and carefully validated experimental modalities such as focused ultrasound/sonogenetics, while maintaining rigorous long-term safety surveillance and addressing ethics, cost, and access to support sustainable translation.
